# Power Consumption of Picture Archiving and Communication System (PACS) Workstations: Quantification and Comparison of Operational Modes

**DOI:** 10.7759/cureus.116698

**Published:** 2026-09-22

**Authors:** Anna Luisa Kühn, Domenic Mongillo, Bilal El Kaddouri, Seyedeh Panid Madani, Daria Kifjak, Alexander A Bankier

**Affiliations:** 1 Department of Radiology, University of Massachusetts Chan Medical School, Worcester, USA; 2 Department of Biomedical Imaging and Image-Guided Therapy, Medical University of Vienna, Vienna, AUT

**Keywords:** energy, hospital sustainability, picture archiving and communication system (pacs), radiology, workstation

## Abstract

Purpose: This study aimed to quantify the power consumption of Picture Archiving and Communication System (PACS) workstations across common operating modes.

Materials and methods: Power consumption was measured for three PACS workstation setups (two hospital-based, one home-based) across five operating modes, including active, standby, powered-down, and transition states. Measurements were conducted using an external power meter, with three repeated measurements per mode and setting (total N = 45). Static modes were extrapolated from 10-minute intervals; dynamic modes included measured duration. Statistical analysis assessed variability and differences between settings. Departmental consumption was estimated by scaling per-workstation data to 105 units and benchmarked against common household appliances. Conversion ratios and break-even times for transitioning between modes were calculated.

Results: Measurement variability was low, with no significant differences between repetitions or inclusion of mammography workstations. At the departmental level, the total consumption reached 24.99 kW in full operation. Even non-active states had considerable power consumption (18.37 kW logged off; 2.41 kW powered down). Mode comparisons showed up to 7.61-fold differences in power consumption. Break-even times were 6.67 minutes (logged-off to active) and 5.86 minutes (powered-down to active), indicating short idle periods justify reduced-power states.

Conclusion: PACS workstations represent a substantial and likely underrecognized source of power consumption in radiology. Quantitative insights and simple decision tools, such as the conversion factors and break-even times presented in this study, can support more energy-efficient workstation management and reduce the environmental impact of radiology operations.

## Introduction

Like many devices used in radiology, Picture Archiving and Communication System (PACS) workstations consume electrical energy [1-3]. The amount of electrical energy consumed by individual PACS workstations is undoubtedly smaller than the energy consumed by picture-generating devices, such as CT or MRI scanners [2,4,5]. This could explain why the energy consumption of PACS workstations has received little attention in the recent literature, despite the increased attention to the overall radiology “carbon footprint” [6-9]. The cumulative energy consumption of PACS workstations may, indeed, be a substantial contributor to the overall energy consumption of radiology, merely because of their number and virtually ubiquitous global deployment [10-12].

Testing the above assumption, however, would require a methodological assessment of the power consumption of PACS workstations, which is not available to date. Moreover, such quantitative assessment could also stimulate and inform usage strategies for reducing energy consumption of PACS workstations, both on an individual and a departmental level [13-15]. The aim of our study, therefore, was to quantify the power consumption of PACS workstations in typical clinical operating modes.

## Materials and methods

Study setting

No human subjects were studied, and no protected patient information was assessed. Therefore, no institutional review board (IRB) permission was requested for this study. All measurements reported in this study were performed by two attending radiologists (ALK and AAB) in December 2024 and January 2025.

The power consumption of three different PACS reading workstation settings were measured: 1) a PACS workstation located in the departmental reading room of the cardiothoracic imaging division of our hospital (HP z440 computer, one 23.8” Lenovo Thinkvision® T24i-10 monitor and one 21” Barco MDRC-2221 peripheral monitor, a 24” Barco 2MP MCRC-2224 clinical review monitor, HP mouse, Lenovo Keyboard, Nuance Powermic); 2) a PACS workstation located in the departmental reading room of the mammography division of our hospital (HP z440 computer, Barco 2MP MCRC-2224 clinical review monitor, two 21” Barco MDRC-2221 peripheral monitors, Agfa mammography keypad, Logitech mouse, Lenovo keyboard, Nuance Powermic); and 3) a PACS workstation setup for remote reading and located in one of the radiology attending’s home (HP Z440 workstation, two 23.8” Lenovo Thinkvision® T24i-10 monitors, two 21” Barco Nio Color 3MP monitors (2048 x 1536 pixels) monitors (portrait: 351 x 491 x 64 mm; landscape: 491 x 351 x 64 mm, screen size: 21.3 inches, calibrated luminance: 600 cd/m^2^ with 1050 cd/m^2^ maximum; screen technology: IPS-SFT Color LCD).

In each of these three settings, five operating modes of PACS workstations were assessed. Operating mode 1 represented normal clinical operation, with the workstation fully active during image interpretation and dictation. Operating mode 2 represented an inactive state in which the PC was in sleep mode and the monitors were in standby mode. Operating mode 3 represented a powered-down state in which the PC was completely shut down while the monitors remained in standby mode. Operating mode 4 represented the transition from mode 2 to fully operational mode 1, and operating mode 5 represented the transition from mode 3 to fully operational mode 1. Modes 1-3 were considered “static” operating modes, whereas modes 4 and 5 were considered “dynamic” transition modes. For the static operating modes (modes 1, 2, and 3), electrical energy consumption in kWh was measured over 10-minute time intervals, as determined by a stopwatch of a cellular phone, and later multiplied by 6 to obtain the average power consumption in kW. For the dynamic operating modes (modes 4 and 5), electrical energy consumption in kWh and the duration of each mode were measured, and the average power consumption in kW was calculated. For each of the three settings and for each of the five operating modes, three repeated measurements were performed, on the same day and in sequential manner, resulting in 45 (3 x 3 x 5) measurements.

All measurements were performed using a Suraielec Energy Monitor (Surai Tech, Hong Kong) interposed between the main energy supply and the PACS workstations. PACS workstations used in mammography are subject to dedicated quality regulations [16]. For example, at our institution, it is not allowed to shut down these workstations overnight to allow for the online quality inspections. Therefore, we included the mammography PACS workstation in our overall power consumption measurements and for comparisons with the non-mammography PACS workstations, as well as for the assessment of measurement repeatability. However, they were not included in the final calculations of average power consumption, as this would have reflected a clinically unrealistic scenario.

Data analysis

All data analyses were performed using MedCalc Statistical Software version 22.023 (MedCalc Software Ltd., Ostend, Belgium) and tabulated on Excel spreadsheets (Excel Workbook, Microsoft 365 MSO 16.0.13801.21004, 32-bit). The individual values of the three repeated sets of measurements were plotted to graphically compare longitudinal variability. In addition, the values of the individual measurements were assessed for potentially significant differences between the three settings: 1) reading room, 2) mammography, and 3) home workstation, using a Friedman test [17]. In addition, a Mann-Whitney test was performed to assess individual measurements including and excluding mammography for potentially significant differences [18].

First, we calculated the average power consumption for each of the five operating modes for a given single PACS workstation in each of the three settings.

Second, to determine the departmental PACS-related power consumption, we multiplied the per-workstation power consumption by the number of PACS workstations in our department (N = 105). We also benchmarked the results of the departmental PACS-related power consumption to the consumption of three commonly used household appliances: a) air conditioner, b) 50’’ flat-screen television, and c) electric water boiler. This is a common strategy in energy policy and systems analysis to make informed determinations, so we chose a similar strategy for our own analysis [19,20]. The reference power consumption for these appliances was taken from the literature, estimated as 3 kW for an air conditioner, 0.016 kW for a 50’’ LED television, and 0.603 kW for an electric water heater [21].

Finally, we calculated the ratios of possible departmental PACS-related power consumptions between the five operating modes and computed the conversion factors between these modes. We also calculated the break-even time, defined as the minimum duration a workstation must remain in a lower power mode for the energy saved relative to mode 1 to offset the energy required for the subsequent transition back to mode 1. The break-even time therefore represents the minimum idle duration at which use of a lower power mode becomes energetically advantageous. Two scenarios were assessed: transition from mode 2 to 1 and transition from mode 3 to 1. This involved dividing the energy consumed during the transition in kWh by the difference in power consumption between the corresponding static modes in kW. The formulas used to calculate break-even points for the two scenarios are as follows: Scenario 1: $\text{Break-even time}=\frac{\text{Mode 4 (kWh)}}{\text{Mode 1 (kW)}-\text{Mode 2 (kW)}}$ and Scenario 2: $\text{Break-even time}=\frac{\text{Mode 5 (kWh)}}{\text{Mode 1 (kW)}-\text{Mode 3 (kW)}}$.

## Results

The individual values for the three sets of measurements are shown in Figure 1. The overall flatness of the lines between the measurement values emphasizes the small amount of measurement variability. Indeed, no statistically significant differences were found between the individual measurements performed in the first, second, and third sequential measurements (Friedman test, F(2, 28) = 2.758, p = 0.081). Moreover, no statistically significant differences were found between the individual measurements performed with and without mammography PACS workstations (Mann-Whitney U test, U = 215.50, Z = -0.229, p = 0.818). Therefore, moving forward, individual measurements for each operating mode were averaged, with their standard deviation, and results were expressed accordingly.

**Figure 1:**
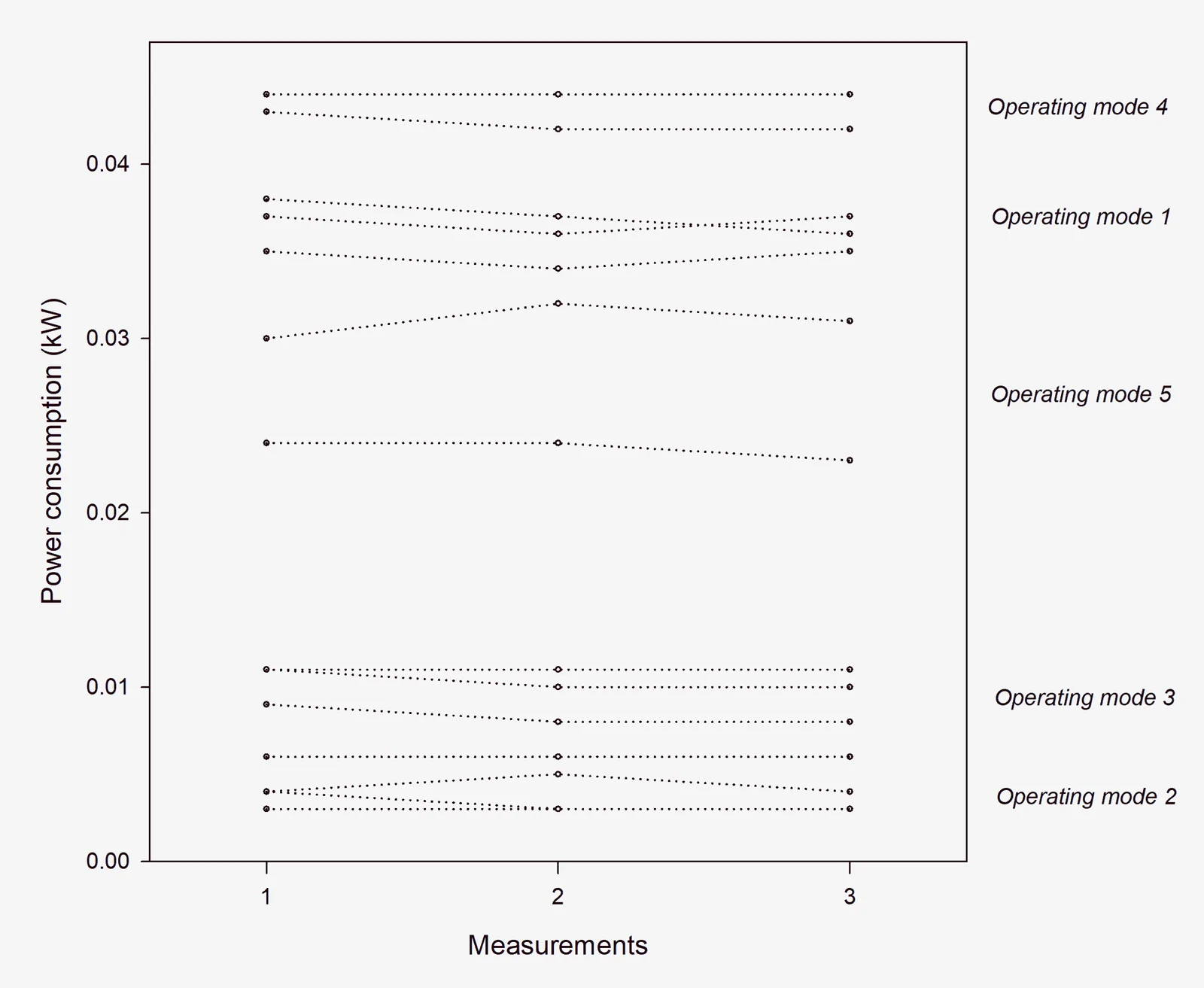
Power consumption measurement variability plot The figure shows all individual datapoints acquired for measurements 1, 2, and 3, respectively. The flatness of the lines for each measurement emphasizes the overall small variability. Of note, only 42 out of 45 measurements are displayed because six measurements were strictly identical and, therefore, graphically completely overlap.

The average power consumption per individual PACS workstation for each of the five operating modes is shown in Table 1. While the overall consumption remains high for dynamic modes 4 and 5, their duration is brief, lasting 1.45 ± 0.05 and 5.69 ± 2.85 minutes, respectively. Using these data, we calculated a break-even time of 6.67 minutes for mode 4 and 5.86 minutes for mode 5. Table 2 shows the calculated power consumption for all 105 PACS workstations of the department, for each of the five operating modes. When fully operational (mode 1), workstations have a total power consumption of 24.99 kW. In addition, they have a consumption of 18.37 kW when the personal computer (PC) is put in “sleep” mode and monitors in “standby” mode (mode 2), 2.41 kW when the PC is shut down and monitors are in “standby” mode (mode 3), as well as 30.45 and 23.21 kW in modes 4 and 5, respectively. This table also benchmarks this departmental PACS-related power consumption to the power consumption of three common household appliances. For example, on a departmental level, one hour of operating mode 1 would be equivalent to 8.33 hours of air conditioning, 1562 hours of watching television (TV), or 41.44 hours of water heating.

**Table 1:** Average power consumption of PACS workstations across five operating modes The table shows the average power consumption per PACS workstation (in kW) for each of the five operating modes assessed in this study, alongside their standard deviation. The table also provides a description of the five operating modes of PACS workstations that were assessed and compared in this study.

	Description	Power consumption (kW)
Operating mode 1	During normal clinical operations, while reporting and dictating (“static” mode)	0.238 ± 0.018
Operating mode 2	PC is in “sleep” and monitors are in “standby” modus. (“static” mode)	0.175 ± 0.036
Operating mode 3	PC is shut down completely and monitors are in “standby” modus. (“static” mode)	0.023 ± 0.005
Operating mode 4	Bringing PACS workstation to “ready for reporting and dictation” status from operating mode 2 (“dynamic” mode)	0.290 ± 0.051
Operating mode 5	Bringing PACS workstation to “ready for reporting and dictation” status from operating mode 3 (“dynamic” mode)	0.221 ± 0.227

**Table 2:** Modelled power consumption of PACS workstations and comparison with household appliance benchmarks The table provides the modelled power consumption of all 105 PACS workstations of our department for each of the five operating modes. Using one hour of PACS operation as the reference period, the corresponding energy use was converted to equivalent operating durations of common household appliances. The benchmark values for these appliances were taken from the literature, which determined to be 3 kW for an air conditioner, 0.016 kW for a 50’’ LED television, and 0.603 kW for an electric water heater [21].

Operating mode	Power consumption for 105 workstations (kW)	Equivalent air-conditioning use (h)	Equivalent TV use (h)	Equivalent water-heating use (h)
Operating mode 1	24.99	8.33	1562	41.44
Operating mode 2	18.37	6.12	1148	30.46
Operating mode 3	2.41	0.8	151	4
Operating mode 4	30.45	10.15	1903	50.5
Operating mode 5	23.21	7.74	1451	38.49

Table 3 shows the ratios of power consumption between operating modes and the resulting conversion factors between the modes. For example, the workstation draws 7.61 times as much power in mode 2 as in mode 3.

**Table 3:** Pairwise comparison of power consumption across PACS workstation operating modes The table shows the comparisons between all operating modes of the PACS workstations assessed in this study. It is aimed at helping radiologists compare power consumptions of the operating modes and make fact-based and informed decisions about the energy-conscious use of their PACS workstations. This chart shows conversions in one direction, but the reciprocals of these calculations are equally accurate.

Comparison of operating modes	Ratio	Conversion factor
1 versus 2	0.238/0.175	1.36
1 versus 3	0.238/0.023	10.35
4 versus 1	0.290/0.238	1.22
1 versus 5	0.238/0.221	1.08
2 versus 3	0.175/0.023	7.61
4 versus 2	0.290/0.175	1.66
5 versus 2	0.221/0.175	1.26
4 versus 3	0.290/0.023	12.61
5 versus 3	0.221/0.023	9.61
4 versus 5	0.290/0.221	1.31

## Discussion

PACS workstations are an omnipresent and undeniable contributor to power consumption in radiology [3,4,22]. Their precise contribution to overall radiology-associated energy consumption, however, is not well known [7-9]. By quantifying power consumption of PACS workstations at the level of a sizeable academic department, our study provides incremental evidence on this topic. Our study also provides comparative data on power consumption of PACS workstations in different operating modes, aimed at supporting individual decision-making about optimizing power consumption in clinical workflows.

Our study confirms that, at the level of our academic department, the power consumption of PACS workstations is substantial. Indeed, our department’s current 105 PACS workstations have a total power consumption of 24.99 kW when fully operational (mode 1), 18.37 kW when logged off (mode 2), and even 2.41 kW when the PCs are fully shut down (mode 3). Despite having no clinical output, mode 2 still consumes the equivalent of 6.12 hours of air conditioning, 1148 hours of TV, or 30.46 hours of water heating. We chose to complement power consumption expressed in kW by benchmarking consumption to that of common household activities (Table 2). Despite inevitably being approximate, given the wide array of different electrical tools available to perform these activities, our approach nonetheless provides an intuitive addition to absolute measures of power consumption. We hope that this information can be helpful to radiologists trying to estimate the power consumption of their PACS workstations on a more general scale.

Our study also shows that power consumption of PACS workstations is non-negligible even when not in fully operational mode, as often occurs during breaks or off-hours. For example, power consumption while all 105 workstations are logged off (mode 2) or even while they are powered down (mode 3) was 18.37 kW and 2.41 kW, respectively. This corresponds to 6.12 and 0.8 hours of air conditioning, 1148 and 151 hours of watching TV, or 30.46 and 4 hours of water heating, respectively. Along similar lines, our study also quantifies the power demand and energy required to bring a PACS workstation up to the fully operational mode from a logged-off or powered-down condition. To compare relative power consumption between operating modes, we have calculated conversion factors between the modes (Table 3). In addition, we calculated break-even time: 6.67 minutes for mode 4 and 5.86 minutes for mode 5. These values show that leaving a workstation in mode 2 for greater than 6.67 minutes would produce enough energy savings to overcome the high energy cost of a transition to active mode 1. Similarly, greater than 5.86 minutes of mode 3 would similarly cover the energy cost of a transition from mode 3 to 1. This is useful for making informed decisions regarding workstation power management. The break-even time may have the potential to support informed and evidence-based decision-making about PACS-related power and energy use in a similar way for individual radiologists in their specific clinical environments.

Our study has several limitations. First, our study was conducted at a single academic medical center. We, therefore, do not know how generalizable our findings are to other medical centers, both in our state and in the U.S. at large. Furthermore, our study was only conducted using three different radiology workstations. As a result, we do not know how generalizable our findings are to workstations in other locations and undergoing different use-cases [23, 24]. In addition, the 10-minute measurement intervals may not fully capture workload-related variations in power consumption during active use, which may depend on the type and number of studies being reviewed. Furthermore, our data collection only consisted of reading workstations. This means our conclusions do not apply to workstations used for other purposes such as administrative or information technology tasks, which have differing use cases and restrictions on operating modes. In addition, some components of the reading workstations studied are identical models, such as the PC itself. This could contribute to the consistency of data from each of the three stations, and affect our final result and the standard deviation for workstation power consumption. As a result, this conclusion once again cannot be generalized to all radiology workstations according to our findings due to their heterogeneity [24]. In summary, our study is faithful to workstations at our institution and represents a valid method of making evidence-based energy policy, but the data and conclusions themselves cannot be extrapolated to other institutions. 

## Conclusions

Our study shows that the power consumption of PACS workstations at the level of an academic department is substantial. Our study also provides intuitive and simple tools that can help radiologists to estimate the power consumption of their PACS workstations and to optimize workstation power management based on quantitative and evidence-based criteria. While radiologists pause and sleep, PACS stations rarely do, as shown by our observations, which may find practical applications and thereby help to reduce the environmental footprint of radiology.
